# Morphology Related Defectiveness in ZnO Luminescence: From Bulk to Nano-Size

**DOI:** 10.3390/nano10101983

**Published:** 2020-10-07

**Authors:** Roberta Crapanzano, Irene Villa, Silvia Mostoni, Massimiliano D’Arienzo, Barbara Di Credico, Mauro Fasoli, Roberto Scotti, Anna Vedda

**Affiliations:** 1 Department of Materials Science, University of Milano-Bicocca, Via R. Cozzi 55, I-20125 Milano, Italy; r.crapanzano2@campus.unimib.it (R.C.); mauro.fasoli@unimib.it (M.F.); anna.vedda@unimib.it (A.V.); 2 Department of Materials Science, INSTM, University of Milano-Bicocca, Via R. Cozzi 55, I-20125 Milano, Italy; silvia.mostoni@unimib.it (S.M.); massimiliano.darienzo@unimib.it (M.D.); barbara.dicredico@unimib.it (B.D.C.); roberto.scotti@unimib.it (R.S.)

**Keywords:** radioluminescence, photoluminescence, defects, excitons, zinc oxide, nanoparticles

## Abstract

This study addresses the relationship between material morphology (size, growth parameters and interfaces) and optical emissions in ZnO through an experimental approach, including the effect of different material dimensions from bulk to nano-size, and different excitations, from optical sources to ionizing radiation. Silica supported ZnO nanoparticles and ligand capped ZnO nanoparticles are synthesized through a sol–gel process and hot injection method, respectively. Their optical properties are investigated by radioluminescence, steady-state and time-resolved photoluminescence, and compared to those of commercial micrometric powders and of a bulk single crystal. The Gaussian spectral reconstruction of all emission spectra highlights the occurrence of the same emission bands for all samples, comprising one ultraviolet excitonic peak and four visible defect-related components, whose relative intensities and time dynamics vary with the material parameters and the measurement conditions. The results demonstrate that a wide range of color outputs can be obtained by tuning synthesis conditions and size of pure ZnO nanoparticles, with favorable consequences for the engineering of optical devices based on this material.

## 1. Introduction

Zinc oxide is a wide band gap semiconductor widely studied since the beginning of the 20th century for various potential applications [1,2,3]. The investigation of ZnO crystals and thin films has attracted great interest in the field of solid-state optoelectronics [1]. Its optical properties, electrical conductivity and radiation hardness make ZnO a good candidate for transparent thin-film transistors, laser diodes, light emitting diodes and photodetectors [2,4,5]. In particular, ZnO is promising for photonic applications in the UV and blue spectral range [3,6] based on exciton emission even at room temperature (RT) because of its wide band gap (~3.4 eV [1]) and large excitonic binding energy (~60 meV [7]), that is about 2.4 times higher than thermal energy (k_B_T) at 298 K. Ferromagnetism at RT has been observed in ZnO doped with transition metals leading to potential application in spintronics devices [3]. Moreover, it is a well-known scintillator [7] and piezoelectric material [8]. 

In recent decades, nanotechnology enabled the possibility of tuning the chemical, morphological, optical, and electrical properties of materials by easy, cost-effective, and low-energy consuming synthesis routes [9,10]. The development of novel nanosized ZnO-based systems promoted both the opportunity of studying stimulated emissions and nonlinear optics in nanometric samples [11], and the exploration of new applications in the field of nanomedicine [12], catalysis [13], photocatalysis [14,15], and gas sensing [16]. In fact, ZnO nanoparticles (NPs) present a high surface reactivity and diverse surface area properties; moreover they are biocompatible and chemically stable [17]. 

Our work takes its grounds on previous optical investigations revealing that, in addition to the near-UV excitonic emission at ~3.4 eV [2], ZnO exhibits several emission bands in the visible (VIS) region of the spectrum, linked to the presence of defect states in the forbidden energy gap [1,7,18]. Both bound and free exciton luminescence have been observed by studying the photoluminescence (PL) temperature dependence of the ZnO UV peak [19]. The bound excitons intensity emission decreases as temperature increases and it disappears between 50 and 150 K [3], whereas free excitons are stable up to RT and their peak position shifts to a lower energy as temperature increases [11]. The ZnO defect emissions in the VIS range have been already investigated, but no consensus has been reached about their origin. The luminescence in the orange–red region of the spectrum is generally detected in oxygen-rich samples: an enhancement of the red emission at ~1.9 eV was observed in ZnO NPs after annealing in air [20] and in ZnO nanorods after SF_6_ plasma treatment [21]. The yellow–green band, reported between 2.2 and 2.4 eV, is the most commonly observed [18] and the most controversial. Its origin has been attributed to singly ionized oxygen vacancies [22], due to the wide consensus of the electron paramagnetic resonance (EPR) signal g=1.96 assignment to these defects [23]. Nevertheless this association is still under debate [24,25,26]. Several others hypotheses have been proposed, among which a recombination mechanism that involves electrons trapped in shallow levels with holes trapped in deep levels [27], and surface defects [28]. Indeed, our previous work on SiO_2_/ZnO systems suggests a correlation between the ZnO green luminescence and surface defects [29]. In addition, it is noticed that in a ZnO single crystal grown by the hydrothermal method, various metal impurities (Li, K, Al, Fe, Pt, Cu), might derive from solution in the crucible [30] and they can introduce energy levels in the ZnO forbidden energy gap [31,32]. Hence, in hydrothermal crystals, the yellow–green band has been attributed to Li ions, whose concentration among the metal impurities is generally the highest [1,2]. Emissions in the blue (~2.6 eV) and violet (~3.0 eV) regions of the spectrum are usually associated to zinc interstitials (Zn_i_) and/or vacancies (V_Zn_) [11]: the former is a shallow donor that lies at ~0.3 eV below the conduction band, while the latter is a shallow acceptor that lies at ~0.3 eV above the valence band [2]. The violet emission has been attributed almost unambiguously to the radiative decay of photogenerated electrons from the Zn_i_ levels and to the valence band [33]. Differently, two mechanisms have been proposed for the blue luminescence: it could occur either due to radiative recombination of electrons between the Zn_i_ and the V_Zn_ levels [34] or between extended Zn_i_ states, which are deeper than simple Zn_i_ states, and the valence band [35]. 

As observed for other nanomaterials [36,37,38,39], ZnO optical properties have demonstrated a high sensitiveness to several features, such as morphology [11,29,40], growth parameters and precursors [41,42], as well as environmental conditions [43,44]. All these parameters concur in defining the defectiveness, that is the concentration and types of point defects present in the material, of ZnO and the resultant luminescence. However, the role of ZnO morphological and surface features has still to be unveiled and its comprehension represents a key point to promote the development of ZnO nanostructures with suitable optical properties.

To provide a significant contribution in this matter, we present a detailed study on the influence of point defects on the optical properties of ZnO whose significance may go beyond the analyzed material itself. In fact, we propose a systematic approach based on the scaling down of the material size, accompanied also by significant changes of growth parameters and interface properties: the comparison between the optical properties of nanometric systems and bulk crystal allows to evidence the emission similarities, due to their same compositions, and to distinguish the luminescence changes imputable to the size parameters. 

Different ZnO particle dimensions, growth conditions, structure and composition of the interfaces are herein examined. In detail, various ZnO systems having different particles size were analyzed: a commercial bulk single crystal (BC), a micrometric commercial powder (MP) and NPs with diameters of 5 nm and 22 nm (N05 and N22, respectively). These latter samples were obtained exploiting two different synthetic strategies: the hot injection process (HI) and the sol–gel method (SG). The optical emissions of all these materials were carefully investigated under both ionizing irradiation (X-rays) and light excitation. Steady state PL and time-resolved (TR) PL were studied as well as the room and low temperature radioluminescence (RL) spectral components, focusing on both exciton and defects emissions.

Interesting correlations between luminescence, sample morphology and point defects in ZnO systems have been outlined, suggesting that the present investigation offers a solid methodological approach, i.e., a systematic spectroscopic analysis scaling from bulk to nanometric size, for an effective assessment of the role of inorganic nanomaterials defectiveness on their optical properties.

## 2. Materials and Methods

### 2.1. Materials Preparation

The investigation is focused on four sets of ZnO, comprising two types of nanoparticles, one commercial micrometric powder and an industrial bulk single crystal. Nanometric ZnO NPs of 5 nm diameter were prepared by a SG method and anchored onto the surface of two silica substrates: either spherical (SS) or rod-like (SR) SiO2 NPs (N05-SG-SS and N05-SG-SR, respectively); while ligand capped ZnO NPs of 5 and 22 nm diameter were synthesized through a HI method (N05-HI and N22-HI, respectively, surface ligand is represented by stearic acid). Moreover, a commercial micrometric ZnO powder’s (MP) dimensions ranging from 100 nm to 2 μm and an industrial ZnO bulk single crystal with hexagonal wurzite structure were analyzed for comparison. A schematic description of the samples is provided in the Table S1 in the Supporting Information.

SiO_2_/ZnO were prepared by a two-step procedure, according to ref. [29]. First, an SG method reported elsewhere [45,46] was exploited to synthesize shape-controlled silica NPs, i.e., SS or SR having different aspect ratios (1, 5, respectively). Successively, silica NPs were used as suitable matrix to grow ZnO NPs (4–6 nm) through a method reported [47,48], in which the hydrolysis and condensation of zinc acetate dihydrate is carried out to get homogeneously distributed ZnO NPs anchored onto the surface of the two supports (N05-SG-SS and N05-SG-SR, respectively). All the reagents were purchased from Merck Life Science (Merck KGaA, Darmstadt, Germany). The reactant amounts were chosen in order to get a nominal ZnO loading on silica equal to 12 wt%. 

ZnO nanometric powder were synthesized using a standard air-free Schlenk technique. In a three necks flask (25 mL), solution A was prepared by dissolving 1 mM of zinc acetate dihydrate (Zn(CH_3_COO)_2_*2H_2_O, ≥ 98%), 4 mM of stearic acid (SA) (C_18_H_36_O_2_, 95%) and 10 mM of 1,2-dodecanediol (CH_3_(CH_2_)_9_CH(OH)CH_2_OH, 90%) in 20 mL of 1-octadecene (90% tech.). In another flask, 4 mM of octadecylamine (ODA) (C_18_H_39_N, 90% tech.,) were dissolved in 4 mL of 1-octadecene (90% tech) (solution B). Both the solutions were degassed at 100 °C for 30 min under vigorous stirring, and two clear solutions were obtained. SA and ODA were employed as capping agents with a molar ratio 1:1, as optimal molar ratio to get homogeneously shaped and sized ZnO NPs [49,50]. All the reagents were purchased from Merck Life Science (Merck KGaA, Darmstadt, Germany) and used as received.

Solution A was then heated up to reflux (270 °C) under a nitrogen atmosphere, until its coloring turned orange. Under reflux conditions, solution B was quickly added to solution A through a syringe (hot injection method) and left under stirring at 270 °C for a suitable time (5 min for N05-HI, 20 min for N22-HI). Then, the mixture was cooled down to RT and the NPs were separated by adding acetone as antisolvent through centrifugation (octadecene:acetone volume ratio equal to 1:1). At last, the solid precipitate was washed twice with fresh hexane (5 mL) and again reprecipitate with acetone (hexane:acetone volume ratio 1:1) and eventually stored in hexane (5 mL).

Preparation of micrometric ZnO powder: Zinc Oxide, Puratronic, ~325 mesh (~44 μm) powder with a purity of 99.999% (metal basis) was purchased from Alfa Aesar by Thermo Fisher (Kandel) GmbH, Kandel, Germany. The sample was labelled as MP.

A ZnO single crystal (BC), grown by hydrothermal method, with purity 99.99% (metal basis), orientation 0001 and two sides polished was purchased from Alineason Materials Technology GmbH, Frankfurt am Main, Germany. The crystal dimensions were 10 mm × 1 mm × 1 mm.

### 2.2. Structural and Morphological Characterization

Powder X-ray diffraction (PXRD) patterns were collected with a Rigaku MiniFlex 600 diffractometer with 0.154 nm Cu Kα radiation (Rigaku Corporation, Akishima-shi, Tokyo, Japan). The measurements were performed in the range 30−80° 2θ (2θ step 0.02°, 1° min^−1^ scan rate).

The morphological features of the nanometric samples (N05-SG-SS, N05-SG-SR, N05-HI, N22-HI) were studied through scanning electron microscopy (SEM) and high-resolution transmission electron microscopy (HRTEM). SEM measurements were performed by a VEGA TS5136 XM TESCAN microscope (TESCAN Orsay Holding a.s., Brno, Czech Republic) in a high-vacuum configuration (electron beam excitation = 30 kV, beam current = 25 pA, working distance = 12 mm). SEM analysis was further employed to verify the size of ZnO particles in a MP micrometric sample. Prior to SEM analysis, the samples were gold-sputtered, to increase their conductivity. HRTEM images of ZnO NPs were collected on a FEI Tecnai Transmission Electron Microscope (FEI Company, Hillsboro, OR, USA) equipped with a Gatan OneView camera (Gatan Inc, Warrendale, PA, USA) operating at 200 kV. ZnO NPs were deposited onto carbon coated Cu TEM mesh grids by drop-casting a dilute NPs dispersion in hexane. The size of ZnO NPs was determined by manually measuring 100 particles randomly chosen in each sample.

Fourier transformer infrared resonance (FTIR) was employed to assess the nature of residual capping agents on the surface of N05-HI and N22-HI. The analysis was performed with a Perkin Elmer Spectrum 100 instrument (Perkin Elmer Inc, Waltham, MA, USA) by a single reflection attenuated total reflection (ATR) method (1 cm^−1^ resolution spectra, 650–4000 cm^−1^ region, 16 scans). 

The quantification of surface ligands on N05-HI and N22-HI was performed through a thermogravimetric analysis (TGA). TGA analyses were carried out using a TGA/DCS1 STARe SYSTEM (Mettler Toledo, Columbus, OH, USA) at constant air flux (50 mL min^−1^), measuring the sample weight loss at increase furnace temperatures (temperature range 30–1000 °C), heating rate 10 °C min^−1^, applying two isotherms at 150 °C and 1000 °C both of 15 min. In these conditions a complete SA combustion was achieved.

### 2.3. Spectroscopic Characterization

Steady state PL spectra were measured by a CW Xenon lamp as excitation source (Horiba Jobin Yvon, Edison, NJ, USA) coupled to a double monochromator Jobin-Yvon Gemini 180 with 1200 grooves/mm gratings (Horiba Jobin Yvon, Edison, NJ, USA) and recorded through a nitrogen cooled, back illuminated, UV enhanced, CCD (charge-coupled device) detector coupled to a monochromator Jobin-Yvon Micro HR with a 150 grooves/mm grating (Horiba Jobin Yvon, Edison, NJ, USA). All the spectra, recorded as a function of nanometers, were firstly corrected for the spectral response of the detector and subsequently converted into spectra as a function of energy. Time resolved photoluminescence (TRPL) measurements were carried out in time correlated single photon counting mode using a FLS 980 spectrofluorometer (Edinburgh Instruments Ltd., Livingstone, UK). TRPL spectra of N05 and N22 monitored at 1.8 eV, of N05-HI, N22-HI, and SA at 2.8 eV, of N05-SG-SS at 2.1 eV, of N05-SG-SR at 2.3 eV, and of BC at 2.2 eV have been collected using a pulsed diode light emitting device (EPLED) emitting at 3.6 eV as excitation source with a pulse width of ~950 ps (Edinburgh Instruments Ltd., Livingstone, UK).; while TRPL decay of N05-SG-SR monitored at 2.7 eV and of MP at 2 eV have been recorded exploiting a pulsed diode laser (EPL) emitting at 3.1 eV with pulse width of ~75 ps (Edinburgh Instruments Ltd., Livingstone, United Kindom). 

Steady state room temperature RL measurements were carried at using a homemade apparatus featuring, as a detection system, CCD Jobin-Yvon Syncerity (Horiba Jobin Yvon SAS, Palaiseau, France) coupled with a spectrograph Jobin-Yvon CP140-1825 operating in the 200–1100 nm range (Horiba Jobin Yvon SAS, Palaiseau, France)All spectra are detected at a pressure of 10^−3^ mbar to avoid the radioluminescence from molecules present in the air and corrected for the spectral response of the detection system. RL excitation was obtained by X-ray irradiation through a Be window, using a Philips 2274 X-ray tube (Koninklijke Philips N.V., Amsterdam, Netherlands) with tungsten target operated at 20 kV and 20 mA. At this operating voltage, a continuous X-ray spectrum with a mean energy of 6–7 keV is produced by a bremsstrahlung mechanism due to the impact of electrons generated through thermionic effect and accelerated onto a tungsten target. The dose rate evaluated on quartz grain matrix is 9.97 ± 0.35 mGy mA^−1^s^−1^ at 20 kV. RL measurements as a function of the temperature were carried out in the 10–300 K interval by using the same instrumentation and a closed-cycle He CTI cryocooler (Helix Technology Corporation, Mansfield, MA, USA), whose working conditions require an ultra-high vacuum (10^−7^ mbar). Only for N05-SG-SS, N05-SG-SR and N05-HI, RL measurements, as a function of the temperature, were carried out exploiting a different detection system made by a CCD Jobin-Yvon Spectrum One 3000 (Horiba Jobin Yvon, Edison, NJ, USA) coupled with a spectrograph Jobin-Yvon Triax 180 operating in the 200–1100 nm range (Horiba Jobin Yvon, Edison, NJ, USA). All the spectra, recorded as a function of nanometers, were firstly corrected for the spectral response of the detector and subsequently converted into spectra as a function of energy.

## 3. Results

### 3.1. Structural and Morphological Properties

The structural features of both micrometric and nanometric ZnO materials were analyzed by PXRD. For all samples, PXRD spectra (Figure S1 in Supporting Information) indicated the presence of the hexagonal ZnO wurtzite crystal phase (JCPDS no. 36–1451). The lower relative intensity and higher width of the (100) and (101) reflections of the nanometric samples prepared by the HI method (N05-HI, N22-HI) supported the nanometric average size of crystalline ZnO NPs, as opposed to the sharp peaks obtained for the microcrystalline MP sample. The size of ZnO NPs determined by the Scherrer equation was equal (8.9 ± 1.3) nm and (15.9 ± 2.4) nm for N05-HI and N22-HI, respectively, in agreement with the average diameters of NPs estimated by TEM images. Besides, the SG procedure further promoted the reduction in the relative intensity of (100) and (101) ZnO reflections in N05-SG-SS and N05-SG-SR, probably due to a slightly lower crystallization degree of ZnO NPs, particularly evident for N05-SG-SS. Finally, PXDR spectra of N05-SG-SS and N05-SG-SR showed an additional broad peak at 2-theta = 22° connected to amorphous SiO2 NPs. The morphology of ZnO particles in all samples was investigated by a combined SEM and HRTEM approach. First, the SEM micrographs of MP (Figure S2) disclose the presence of micrometric agglomerates of particles very inhomogeneous both in shape and dimensions, which range from 100 nm to 2 μm.

**Figure 1:**
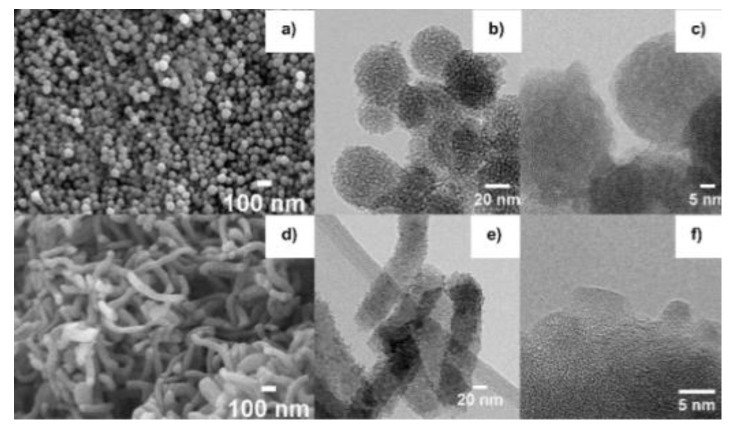
SEM and HRTEM images of N05-SG-SS (**a**–**c**) and N05-SG-SR (**d**–f), respectively. SS: spherical, SG: sol–gel method, SR: rod-like.

**Figure 2:**
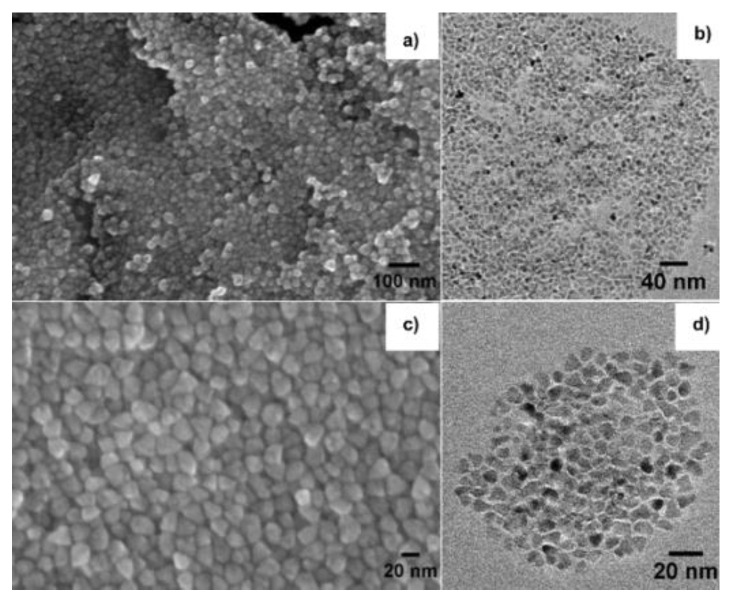
SEM images of N22- hot injection process (HI) (**a**,**c**) and high-resolution transmission electron microscopy (HRTEM) of N05-HI (**b**,**d**).

SEM and HRTEM images of N05-SG-SS and N05-SG-SR were consistent with our previous results [29,45,46] and corroborated that, through this optimized synthetic procedure, nanometric ZnO NPs (~5 nm) were anchored onto the surface of porous SiO_2_ NPs having different aspect ratios (average diameter of SiO_2_ spheres ~30 nm in N05-SG-SS, average length of SiO2 rod-like particles ~400 nm in N05-SG-SR, Figure 1). HRTEM and SEM confirmed the nanometric size of N05-HI and N22-HI, by showing almost spherical NPs with a size of (5 ± 1) nm and (22 ± 5) nm, respectively (Figure 2). 

Thus, in these experimental conditions, the HI method allowed the synthesis of differently sized ZnO NPs by simply changing the reaction time (equal to 5 and 20 min for N05-HI and N22-HI, respectively), even though higher reaction times were accompanied by a lower control of the NPs shape (Figure 2a,c). In this synthetic procedure, stearic acid and octadecylamine guaranteed in both cases a fine control on the nucleation and growth process of ZnO NPs, hindering their further growth towards the micro-crystalline scale by behaving as capping agents to ZnO NPs. FTIR spectra, collected for N05-HI and N22-HI, confirmed the presence of SA molecules as main ligands on the surface of ZnO NPs (Figure S3), as demonstrated by: (i) the asymmetric and symmetric stretching of CH_2_ groups at 2916 and 2860 cm^−1^, (ii) the asymmetric and symmetric stretching of the carboxylate groups (-COO-) at 1538 and 1398 cm^−1^, typical of the zinc stearate structure, that corresponds in this case to SA molecules coordinated to Zn atoms onto the surface of ZnO NPs. SA quantification in N05-HI and N22-HI was performed by TGA as reported in Figure S4. Based on TGA outputs, the amount of SA organic ligand, corresponding to the weight loss measured by TGA between 150 and 1000 °C (ΔW_150–1000 °C_), was 80 wt% for N05-HI and 20 wt% for N22-HI, in agreement with the higher surface/volume ratio and surface energy of ZnO NPs in N05-HI, compared to N22-HI.

### 3.2. Radio- and Photo-Luminescence

The luminescence properties of all ZnO samples were investigated at RT under both ionizing radiation and optical excitation. For the former analysis, we exploited irradiation by low energy X-rays; measurements were performed in rotary vacuum condition (10^−3^ mbar) to avoid the RL signal caused by air molecules ionization. For photoluminescence, we selected the excitation energy from a continuous wave (CW) Xenon lamp. Each sample revealed peculiar optical features with emissions from defects and/or excitonic states. 

**Figure 3:**
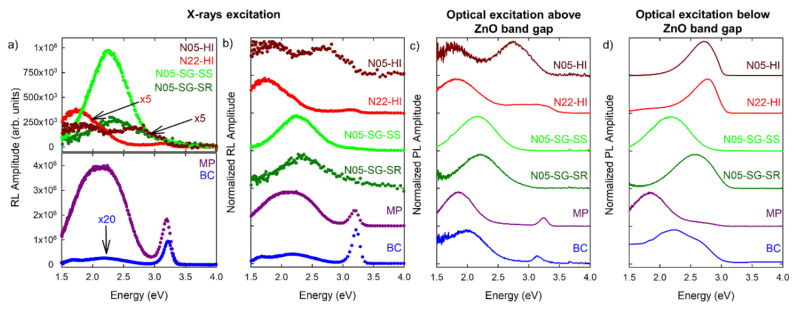
Optical properties of different ZnO materials: (**a**,**b**) Room temperature (RT) radioluminescence (RL) intensities and normalized RL spectra under X-ray irradiation, respectively; for all samples the spectra are measured at 300 K and 10^−3^ mbar pressure, operating the X-ray tube at 20 kV and 20 mA; (**c**) Normalized photoluminescence (PL) spectra of all samples recorded under continuous wave (CW) excitation at 3.5–3.6 eV (above ZnO band gap) with the use of a long-pass filter with cutting wavelength at 370 nm (3.4 eV); (**d**) Normalized PL spectra of all samples recorded under CW excitation at 3.1–3.2 eV (below ZnO band gap) with the use of a long-pass filter with cutting wavelength at 418 nm (3.0 eV).

Figure 3a shows the absolute RL intensities of the nanometric samples, the micrometric commercial specimen, together with the bulk crystal. We remark that the BC is transparent and yellowish at variance with the other samples which are white powders (Figure S5). All the spectra were collected under the same experimental conditions and normalized to the ZnO weight percent in order to allow an absolute comparison among their amplitudes. The overall emissions of the nanometric powders are about 10 and 70 times weaker than that of the micrometric one for the silica supported N05-SG-SS and N05-SG-SR NPs and for the bare N05-HI and N22-HI NPs, respectively. The RL efficiency of the bulk sample is about 250 times weaker with respect to that of the micrometric powders. In this regard, it has to be noticed that the comparison between crystal and powders must take into account the effect of different morphologies on light collection: the detection of light emitted from crystal can be affected by the waveguide effect [51], differently than in highly scattering white powder systems, in which the luminescence is more effectively conveyed into the collection angle. 

Figure 3b displays the RL spectra normalized to their maximum. The relative intensity of the UV exciton emission with respect to the defects bands decreases by scaling down the material size. In fact, the excitonic luminescence is clearly revealed in the crystal, it is still evident in the micrometric powder, while it is very weak or absent in the nanometric samples. The shape of VIS defect-related luminescence varies throughout the specimens: BC and MP samples present broad composite emissions extending from 1.5 eV to almost 2.9 eV, while the nanometric systems display distinct peaks in the green (N05-SG-SS and N05-SG-SR), red (N05-HI and N22-HI) and blue (N05-HI) region of the spectrum. A detailed analysis of the RL spectral components of all the samples emissions is carried out in the following Section. The absolute RL spectra of bare silica NPs (Figure S6a), exploited as substrate for N05-SG-SS and N05-SG-SR systems, and of the stearic acid (Figure S6b), anchored to the surface of N05-HI and N22-HI samples, display a very weak and absent signal, respectively, proving that the optical properties observed in all the nanometric powders under X-ray excitation are completely attributable to ZnO. 

Figure 3c,d show the normalized PL emission spectra of all ZnO systems acquired under light excitation above (3.5–3.6 eV) and below (3.1–3.2 eV) the ZnO band gap, with the use of a long-pass filter with a cutting wavelength at 370 nm (3.4 eV) and at 418 nm (3.0 eV), respectively. The exciton luminescence, excited only above ZnO band gap, is detected solely in BC and in MP and the ratio between its intensity and those of the defect emissions is lower with respect to the one under X-ray excitation (Figure 3c). The defect-related emission of each sample is analogous to the RL one observed when the excitation energy is higher than the ZnO band gap. Instead, modifications in the spectral shape are observed when exciting below the ZnO band gap, with the exception of the N05-SG-SS emission (Figure 3d). BC and MP systems exhibit a slight change in the PL spectra, due to an enhancement of the emission intensities in the green and blue region of the spectrum. Differently, three nanometric specimens (N05-SG-SR, N05-HI and N22-HI) show a significant variation of their luminescence, due to the presence of a bright blue luminescence. To complete the spectroscopic analysis of the nanometric samples, PL properties of the silica substrates, and of the capping agent were investigated. The PL measurements evidence that the SS and SR are non-luminescent (Figure S7a), while SA shows an emission peaked at around 2.8 eV that overlaps the bright luminescence observed in N05-HI and N22-HI when these NPs are excited at energies lower than the ZnO band gap (Figure S7b). Consequently, further analyses are necessary to unequivocally assign the origin of this blue component either to defect species or to the capping agent.

### 3.3. Analysis of Spectral Components

RL measurements as a function of temperature were carried out from 10 to 300 K on all the ZnO systems to further investigate the features of exciton and defect emissions. The use of a closed-cycle He Cryogenic Technologies Inc. (CTI) cryo-cooler requires high vacuum conditions (10^−7^ mbar), differently than in the previous RL experimental procedure in which higher pressures (10^−3^ mbar) were adequate. Nanometric N05-SG-SS and MP samples display a pressure-sensitive behavior (Figure S8). Lowering the pressure from 10^−3^ mbar to 10^−7^ mbar, an increase in the exciton luminescence intensity with respect to the defect one is observed in the micrometric MP specimen. Moreover, the RL spectral shape of N05-SG-SS is also slightly affected by the change in pressure, since an additional broad emission, extending from approximately 2.5 eV to 3.2 eV, is detected under high vacuum conditions. 

**Figure 4:**
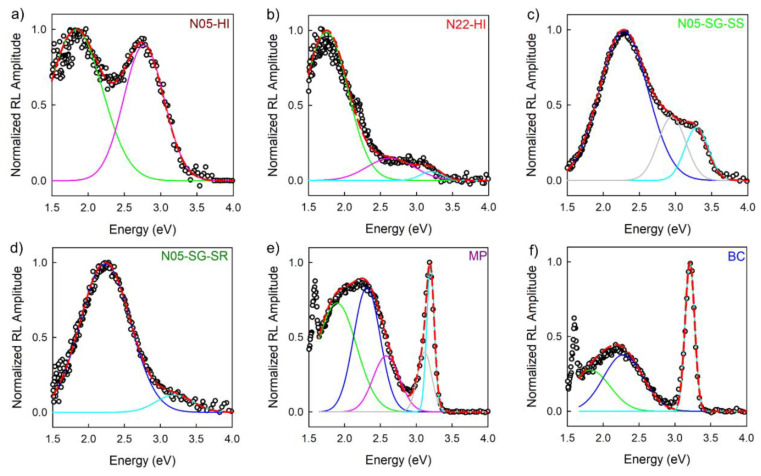
Gaussian deconvolution of normalized RL spectra of different ZnO materials at 300 K and 10^−7^ mbar (**a**–**f**): Gaussian components (green, blue, pink, light blue, and grey solid lines) obtained by numerical fit are shown together with experimental curves (black empty circle lines). The curve representing the whole numerical fit (red dashed line) is superimposed to the experimental data. The sets of all parameters of the deconvolution are listed in Table S2.

Figure 4 displays the Gaussian deconvolutions of the room temperature RL spectra of all the ZnO samples: overall five bands were identified, one related to excitonic transition and four to defect centers. The outcome of the numerical analysis shows that the UV excitonic emission, labelled as band E, is centerd at 3.27 eV with an uncertainty of 4%, whereas the VIS defect-related luminescence components are peaked in the red (band A centerd at 1.83 eV with an uncertainty of 3%), green (band B centred at 2.29 eV with an uncertainty of 6%), blue (band C centerd at 2.68 eV with an uncertainty of 4%), and violet (band D centerd at 3.05 eV with an uncertainty of 7%) region of the spectrum. The set of all the parameters of the numerical analysis, together with their percentage weight at 300 K, are reported in Table S2 in the Supporting Information.

Nanometric powders synthesized through the same route share similar spectral components, disclosing nevertheless slight differences. The bare N05-HI and N22-HI NPs exhibit the same defect bands, A and C, while only in N22-HI the excitonic one, labelled as E, is present (Figure 4a,b). As already mentioned, the RL emissions of N05-SG-SS recorded at 10^−3^ mbar and at 10^−7^ mbar (Figure S8) differ from each other: the in rotary vacuum condition, two spectral components, B and E, were identified (Figure S9), while in the high vacuum one an additional band, D, appears (Figure 4c). The spectrum of N05-SG-SR is similar to the N05-SG-SS one at 10^−3^ mbar (Figure 4d). Differently, all the five spectral components were necessary to reconstruct the RL emission of the micrometric powder, as shown in Figure 4e. In the bulk crystal, two defect spectral components, A and B, and the exciton one (E) were recognized (Figure 4f). The peak at 1.6 eV observed in the bulk and micrometric sample is just the second order of the exciton emission. Furthermore, for all the samples, PL spectra, collected both under excitation above and below the ZnO band gap, were reconstructed by a Gaussian deconvolution procedure (Figures S10 and S11, Tables S3 and S4). Interestingly, five components, corresponding exactly to the bands identified in the RL emissions, were required for satisfactory spectra reproduction. Slight discrepancies in the energy peak positions and in the Full Width at Half Maximum (FWHM) values can be due to the different detection system employed in the RL and PL measurements.

**Figure 5:**
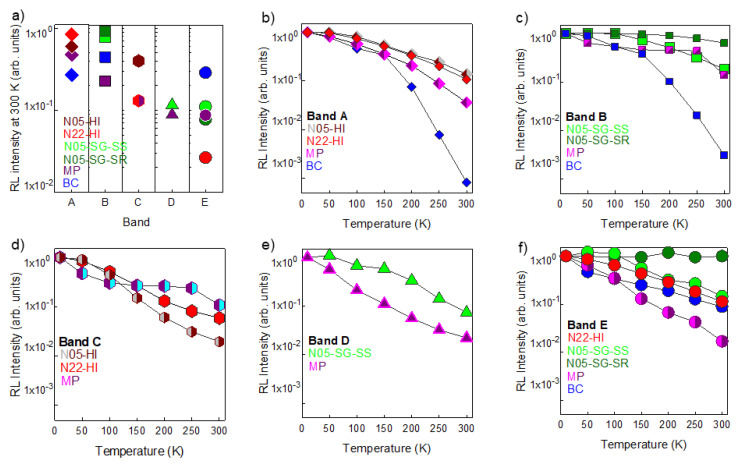
Temperature influence on RL spectral components of different ZnO materials: (**a**) Integrated RL intensities, displayed on a logarithmic scale, at 300 K of band A (diamond), B (square), C (hexagon), D (triangle), E (circle). For each sample, the integrated RL of a single component is normalized to the total RL intensity at 300 K. (**b**–**f**) Integrated RL, displayed on a logarithmic scale, versus temperature of the five spectral components. For each component, the integrated RL is normalized to its integrated RL recorded at 300 K.

Low temperature RL spectra disclose that the overall light output decreases as the temperature increases, due to thermal quenching, in all ZnO samples except for N05-SG-SR, whose emission efficiency is almost constant (Figure S12). For all ZnO systems, the evolution of their RL properties was investigated by performing the Gaussian deconvolutions of spectra recorded at different temperatures. The results of the fit procedure disclose the presence, in each sample, of the same bands detected at 300 K (Figure 5). Figure 5a shows for all the ZnO systems the integrated RL of each component, normalized to the area of the total light output at RT. Figure 5b–f display the integrated RL, normalized to their value at 10 K and as a function of temperature, of the five bands. In Figure 5, the RL intensities are displayed in logarithmic scale.

The obtained data enlighten a stronger temperature dependence in the bulk and micrometric sample luminescence with respect to the nanometric powders. We estimated the thermal activation energy [52] of the exciton luminescence for all the three systems (bulk, micrometric powder and NPs) and of band A, B, and C for the nanometric samples: our findings, reported together with the used procedure in the Supporting Information are in agreement with the literature [53,54,55,56]. Besides the luminescence intensity, the RL spectral shape of BC, N05-SG-SS and N05-HI also change as a function of temperature (Figure S12) and at 10 K the defect bands, in the green–blue region of the VIS spectrum, are dominant. Moreover, Figure S12 discloses that the excitonic peak (band E) shifts its position towards higher energies and gets broader as the temperature increases, in accordance with the suppression of bound excitons and the characteristics of the free ones [3,11].

### 3.4. Time Resolved Luminescence

**Figure 6:**
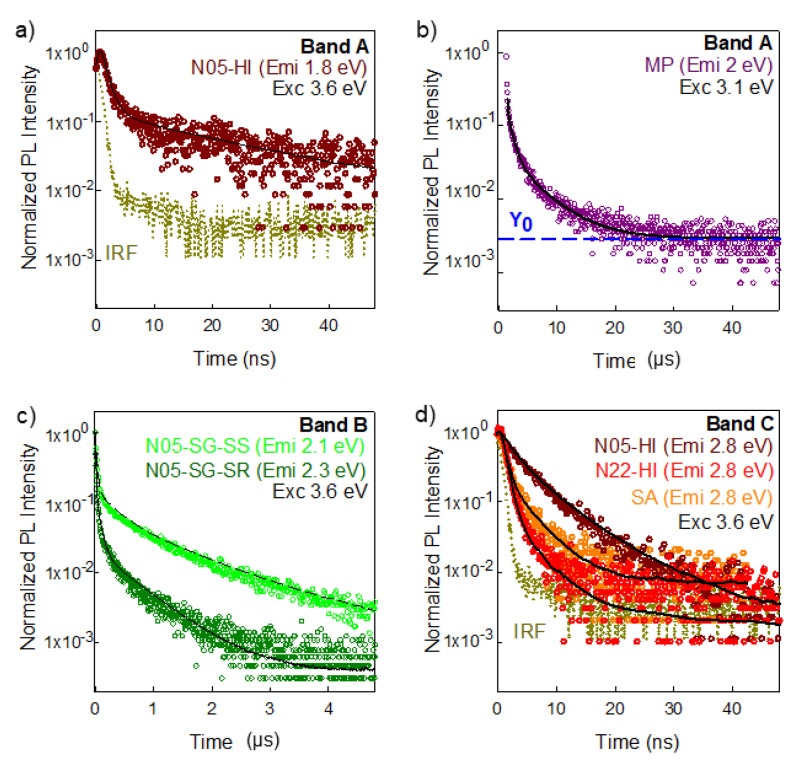
Time dynamics of excitonic and defect emissions in different ZnO materials: (**a**) PL time decays of one bare ZnO NPs (N05-HI) at 1.8 eV (corresponding to band A) under pulsed excitation at 3.6 eV; (**b**) PL time decays of micrometric powder (MP) at 2 eV (corresponding to band A) under pulsed excitation at 3.1 eV (**c**) PL time decays of ZnO NPs grown on silica substrates (N05-SG-SS, N05-SG-SR) at 2.1 eV and 2.3 eV, respectively (corresponding to band B), under pulsed excitation at 3.6 eV; (**d**) PL time decays of the bare ZnO NPs (N05-HI, N22-HI), and their capping agent (stearic acid, SA) at 2.8 eV (corresponding to band C) under pulsed excitation at 3.6 eV. In panels (**a**,**d**), the instrument response functions (IRF) are also shown (mustard dotted line). From (**a**–**d**), the signal decays are fitted as multiexponential functions (black solid lines). In (**b**) a constant parameter (y0) as backgrounds was also used. The sets of all parameters used to model the PL decay are reported in Table S5.

ZnO optical properties were further investigated by the analysis of the time decay of the bands identified by the Gaussian deconvolution of the emission spectra. In all the ZnO samples that display the exciton luminescence (corresponding to band E), we tentatively measured its lifetime, observing that it is faster than the instrumental response (tens of picoseconds). This finding indicates that the time decay of the ZnO excitonic band is in the sub-nanosecond range, in agreement with the literature [1]. For the ZnO VIS defect-related emissions, we focused the TRPL measurements on the samples in which a specific band was clearly distinguishable, in order to ensure a selective collection of the signals originated from different centers. Taking into account the results of the PL deconvolution (Figures S10 and S11), we chose to investigate the time dynamics of the red luminescence (band A) of the bare N05-HI, N22-HI NPs and of MP, and we selected N05-SG-SS, N05-SG-SR nanosystems for the analysis of the TRPL signal of the green emission (band B). Differently, the study of the time decay of the blue component (band C) was performed on N05-HI, N22-HI samples and, for comparison, on their capping agent. Lastly, the time dynamics of the violet emission (band D) was not investigated, since the strong overlap of this band with the others prevents the exclusive collection of its signal. The TRPL signals of the red luminescence (band A) were collected at 1.8 eV (exciting at 3.6 eV) and at 2 eV (exciting at 3.1 eV) for nanometric N05-HI and N22-HI NPs and for MP, respectively. Since exciting both at higher and lower energies than the ZnO band gap, the defect emission of the MP sample is similar (Figure 3c,d), the experimental condition with the better signal to noise ratio was chosen. Very interestingly, the fastest decay of the red spectral component (band A) was observed in the smallest ZnO systems: the performed multi-exponential fit discloses average lifetimes of the order of tens and thousands of nanoseconds, for N05-HI, N22-HI and for MP samples, respectively (Figure 6a,b and Figure S13, Table S5). Moreover, to successfully reconstruct the TRPL signal of the MP, a constant background was also added: this result hints that the time dynamic of the recorded emission is even slower. To study the decay of the green luminescence (band B), TRPL measurements were performed on N05-SG-SS and N05-SG-SR, recording the emission at 2.1 and 2.3 eV, respectively. Their decays are fitted with three-exponential functions (Figure 6c, Table S5) and the average lifetimes are of the order of hundreds of nanoseconds and are shorter for N05-SG-SR than for N05-SG-SS. For the blue emission (band C), the TRPL signal of bare N05-HI, N22-HI NPs and their capping agent was collected under excitation at 3.6 eV at the PL maximum intensity peaked at 2.8 eV. Time decays of the two ZnO samples both fitted by a bi-exponential curve differ, resulting in average lifetimes of the order of few of nanoseconds, faster for N22-HI than for N05-HI (Figure 6d, Table S5). Notably, the time decay of SA and of N22-HI are similar (Figure 6d, Table S5).

Hence, taking into account the RL and PL features of these two specimens, we hypothesized that the emission peak at 2.8 eV originates from defect centers in N05-HI, while it is mainly due to the stearic acid in N22-HI. In fact, under X-ray excitation, the capping agent is not luminescent (Figure S6b) and the blue spectral component (band C), detected both in N05-HI and N22-HI, is more intense in the smallest NPs (Figure 4a,b), suggesting that the concentration of the defect species emitting at 2.8 eV is higher in N05-HI than in N22-HI. These observations are in agreement with TRPL measurements. In N05-HI, because of the occurrence of a high content of defect centers emitting at 2.8 eV, their emission prevails and consequently its decay time is different from that of SA. On the other hand, due to the lower concentration of blue emitting defect species in N22-HI, the SA emission overcomes the one from ZnO, as also the similar time dynamics of N22-HI and SA validate. For all the multi-exponential PL decays, the complete data set of the obtained decay components with corresponding weights is reported in Table S5.

## 4. Discussion

Aiming at providing useful guidelines for the analysis and characterization of ZnO nanostructures for applications in photonic and optoelectronic devices, the observed variations in the RL and PL properties of ZnO samples will be discussed in terms of excitation energy exploited in the experiments, particle size, growth conditions, and interfacial features, which have an additional impact on defectiveness and spectroscopic properties.

### 4.1. Role of Excitation Energy

The optical features of all ZnO systems were investigated exploiting three different excitation energies: (1) ~6.5 keV (ionizing radiation mean energy) in RL measurements exciting from deep core bands to high energy electron levels with the production of a cascade of free electrons and holes, (2) 3.5–3.6 eV in the PL ones exciting from valence to the conduction band, and (3) 3.1–3.2 eV in the PL ones exciting from valence band to intragap states in PL ones (Figure 3). RL and PL techniques exploit different excitation sources: ionizing radiation and light, respectively, leading to significantly different excitation mechanisms and, consequently, different relaxation pathways. In RL, X-rays, as a result of the ionization of electrons from the core bands of the analyzed material, are converted into high energy electron–hole pairs that undergo a cascade multiplication of free carriers followed by their thermalization towards the edges of the conduction and valence bands. The generated free carriers can then migrate within the delocalized bands. Different relaxation processes, other than prompt radiative recombination, such as non-radiative energy losses and trapping by traps, can occur. Hence, the RL technique unveils the effect of carriers migration and the role of traps on the sample luminescence, but its high energy excitation, which involves core levels, is not selective towards optically active recombinations, either of excitonic or of defect origin. At variance, in PL measurements, the excitation energy, which is close to or even lower than the energy band gap, can be properly chosen, allowing for the monitoring of transitions between valence bands and intra-gap electronic levels, or between intra-gap states. 

The spectral shapes of the room temperature RL and PL emissions recorded as exciting just above the ZnO band gap are generally similar for all the ZnO samples, suggesting that events of charge carriers trapping on defects are not relevant in the RL carrier migration [57,58]. Interestingly, some differences can be noticed in the relative intensities of excitonic and defect emissions, the former one being more intense under X-ray excitation. In PL measurements, an incident photon generates one electron and one hole, while in RL the high energy radiation is converted into numerous carriers. In both techniques, the free carriers, that move into the material during thermalization, can recombine through defect centers, or form excitons (coupled electron–hole pairs). We propose that in RL the higher concentration of generated charges favors the exciton formation and emission with respect to above band gap light excitation, even in very defective samples. On the other hand, exciting below the ZnO energy gap, luminescence emissions from defect species in the green (band B) and blue (band C) region of the spectrum are enhanced with respect to above gap excitation, especially in nanometric systems. 

### 4.2. Role of Dimensionality

Although we consider any quantum confinement effect in the investigated ZnO nanosystems as negligible, since their dimensions are bigger than the ZnO Bohr radius (~2 nm) [3], several effects related to nanometric dimensions are unveiled. The overall efficiency of luminescence in ZnO is compromised when scaling down the size, as Figure 3a discloses. This observation suggests that non-radiative dissipation of excitation energy is enhanced in NPs as is also corroborated by TRPL measurements. In fact, the defect bands decay dynamics are strongly accelerated with the reduction of dimensions (Figure 6a,b). In micrometric powders, the red luminescence (band A) decay time is at least in the order of microseconds, in agreement with those reported in the literature for defect emission of ZnO crystals and ceramics [1,7]. Instead, the average lifetimes of the same band A of bare N05-HI, N22-HI NPs are of about tens of nanoseconds. Additionally, in previous studies, ZnO nanostructures displayed defect emission complex decay behaviors with fast time dynamics [18,34]. Nanometric systems are characterized by a significant degree of defectiveness mainly because of their high surface-to-volume ratio [59]. Interfaces increase disorder, leading to the formation of surface related defect centers, like dangling bonds, hydroxo or peroxo linkages. The smaller the material, the higher the probability that the excitons reach the surface and interact with its defects, leading to both radiative and non-radiative recombination. On the basis of Monte Carlo simulations, it has been reported that, in spherical ZnO nanocrystals, the percentage of excitations that decay on the surface, and of these, the ones that undergo non radiative annihilation, increase scaling down the size: in particular almost all the generated excitons reach the surface and recombine non radiatively in systems with a radius of a few nanometers [60]. Hence, surface states often act as quenching channels [61], causing a decrease in the decay time of radiative transitions [62]. Consequently, ZnO NPs typically display a time decay acceleration as the size is reduced [18]. 

The ZnO dimensionality also impacts on the intensity ratio between the excitonic (UV) and defect (VIS) emissions (Figure 3). In order to perform a precise analysis, we focused on the room temperature RL spectra and their Gaussian deconvolutions (Figure 4 and Table S2): the highest ratio between the exciton and defect luminescence is observed in the bulk material, and then it diminishes in micrometric powder and becomes even weaker in nanometric ones. Interestingly, only the Gaussian deconvolution of one bare nanometric N05-HI sample does not require the excitonic band to perform an accurate spectrum reconstruction, even if the NPs diameter (5 nm) is the same as that of the ZnO NPs grown on SiO_2_, (Table S2). Our results are in agreement with previous studies in which it has been observed that under X-ray excitation, the ZnO excitonic peak results are more intense than the defect one in mono and poly-crystals, while they are weaker in ceramics and powders [63,64,65]. Our study clearly highlights that the exciton emission decreases by scaling down the size of the system. Since the point defects concentration is enhanced, reducing the dimensions, this result confirms that the intensity of the exciton luminescence is strongly affected by the presence of defect states. Furthermore, the excitonic emission, almost resonant with the ZnO band gap, can be reabsorbed by the sample [66,67], thus increasing the probability of interaction between the excitation and the defects. Our suggested interpretation is that, in nano-systems, the probability that the excitations transfer their energy non-radiatively to emissive defects or quenching centers—rather than undergoing radiative relaxation—is higher than in micrometric and bulk samples, which typically are affected by a lower degree of defectiveness. 

Moreover, RL spectra of the micrometric powder and of a nanometric N05-SG-SS sample display that the intensity ratio between the exciton and the defect peaks increases (Figure S8), lowering the pressure from 10^−3^ mbar to 10^−7^ mbar. Both ZnO UV excitionic and visible defect-related luminescence are indeed pressure and moisture sensitive. In particular, it has been found that the excitonic emission in ZnO quantum dots can be quenched in humid air [43] and it has been observed that annealing in an O_2_ atmosphere suppresses both the exciton and the green emission in ZnO single crystals [68]. Hence, we suggest that in a rotary vacuum the exciton peak is suppressed by gaseous molecules, especially H_2_O adsorbed on the samples surface, whereas in a high vacuum, after their release, it is restored. This interpretation could be supported by the observation (not reported) that the exciton emission of MP is progressively enhanced under continuous X-ray irradiation; indeed, the increase in RL efficiency after prolonged irradiation (bright burn) is a known phenomenon in scintillators and it is generally due to the progressive filling of traps in the forbidden energy gap [69]. Hence, the observed bright burn of the MP exciton peak suggests that its surface states, when they are already occupied by the constantly pumped electrons, cannot act anymore as carrier traps. 

The luminescence efficiency of all bands is decreased by increases in temperature, revealing the occurrence of thermal quenching (Figure S12). The activation of non-radiative multi-phonon relaxation, as a consequence of the temperature increase, is a common mechanism that contributes to this phenomenon [52]. Other processes influence the luminescence temperature dependence, among which the competition between radiative and non-radiative recombination centers, whose effects raise with increases in temperature [70] or the thermally activated depopulation of emissive defect levels near the conduction band edge [71], which allows the excitations to migrate, reach the surface and relax non-radiatively or be re-trapped by other defect centers. It is worth noting that in nanometric systems the exciton emissions are less intense than the defect ones even at low temperatures (Figure S12b); therefore, we conclude that in NPs the radiative recombination at defects is favored also at low temperature, because of the high degree of defectiveness and the small size. Figure 5 discloses that the optical properties of nanometric powders are less affected by temperature variation than those of the micrometric sample and of the single crystal. This supports that the effects of the limitation of carrier’s migration, caused by the decrease in thermal energy, are less significant in nanosystems than in bulk and micrometric materials.

### 4.3. Role of Growth Conditions and Interfaces 

**Figure 7:**
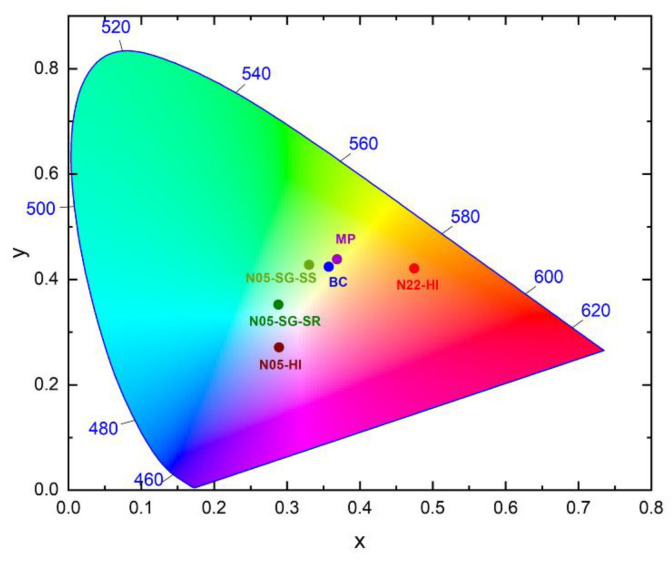
Color output of all ZnO samples: CIE1931 chromaticity diagram reporting the coordinates of RT RL emissions of N05-HI (dark red circle), N22-HI (red circle), N05-SG-SS (green circle), N05-SG-SR (dark green circle), MP (purple circle), and BC (blue circle). Authors should discuss the results and how they can be interpreted in perspective of previous studies and of the working hypotheses. The findings and their implications should be discussed in the broadest context possible. Future research directions may also be highlighted.

Through a Gaussian deconvolution analysis, the RL visible emissions of various ZnO samples were reconstructed exploiting a maximum of four defect bands, all well documented in previous studies [1,11]: band A and B match the red and yellow–green luminescence, respectively, whereas C and D match the blue and violet ones. We recall that the visible emissions are certainly due to intrinsic defect species in the nanometric and micrometric powders, whereas for the single crystal, grown by the hydrothermal method, it is not possible to neglect the presence of metal impurities, especially Li, which can influence the ZnO luminescence [30]. As Figure 4 shows, bands A and B are the more common and intense throughout the samples. Except for N05-HI, bands C and D contribute less to the overall light output but are necessary for an accurate spectrum reproduction. Except for N05-HI, bands C and D contribute less to the overall light output but are necessary for an accurate spectrum reproduction. The same spectral components accompanied by a similar efficiency at RT (Figure 3a) were detected in nanomaterials synthetized by the same procedure, corroborating the influence of growth conditions on the final point defects type and concentration. The important role of morphology and interfaces is further evidenced by the differences in the PL features of ZnO NPs grown on two different silica substrates, having spherical (in N05-SG-SS) or rod-like (in N05-SG-SR) shapes. The occurrence of inequivalent defect species in these samples is disclosed by their different PL spectra—under excitation below the ZnO energy gap: N05-SG-SS exhibits only the green luminescence (band B), whereas the PL spectrum of N05-SG-SR is the convolution of the green (band B), blue (band C), and violet (band D) Gaussian components (Figure S11c,d). Moreover, a higher concentration of quenching centers in N05-SG-SR with respect to N05-SG-SS is revealed by the acceleration of the green emission time dynamics of N05-SG-SR (Figure 6c). Hence, just by using substrates with different morphologies, i.e., spherical (in N05-SG-SS) or rod-like (in N05-SG-SR), the surface properties and, consequently, the optical features are affected. Regarding the isolated NPs, the PL features of the bare N05-HI and N22-HI NPs indicate that the stearic acid anchored on the nanomaterials do not introduce novel defect centers or surface states in ZnO structures. Hence, the ZnO intrinsic defectiveness is not modified, but the ligand itself can be emissive in certain excitation conditions altering the final luminescence of the nanosystems, as also highlighted by TRPL results. Therefore, our findings unveiled the impact of synthesis conditions on the optical properties. Moreover, despite that the NPs crystallinity degree changes significantly with the synthesis process (HI or SG) and the morphology of the silica substrates (Figure S1), striking correlations with the luminescence features were not observed. Further studies will be performed to assess the relationship between the ZnO NPs crystallinity level and optical properties. In Figure 7, the chromaticity diagram created by the Commission internationale de l’éclairage in 1931 (CIE 1931), a powerful visualization tool, reports the coordinates of the RL of all the ZnO samples. It unveils that the emitted light of BC and MP samples are green–yellow, while the color outputs of the nanometric powders cover different regions of the spectrum (green for N05-SG-SS, bright blue for N05-SG-SR, violet for N05-HI, and orange for N22-HI), corroborating the influence of point defects on the luminescent properties.

## 5. Conclusions

In this work, a well-known and versatile semiconductor, such as ZnO, was selected as a model material to conduct a detailed study of the relationship between particle size and interfaces, defectiveness, and optical properties. ZnO luminescence was investigated through a valuable methodological approach involving three sample size (nanometric, micrometric, and bulk) and different excitation sources (ionizing radiation and light). An accurate analysis of the RL spectral components performed on all the samples highlighted the presence of a UV excitonic band, accompanied by four defect-related VIS ones. We also unveiled that the excitonic emission is strongly affected by size: its intensity—with respect to that of the defect luminescence—decreases by reducing the particle size. We clearly evidenced that the point defect concentration is influenced by the particle dimensions and growth conditions, leading to different colored light outputs, luminescence temperature dependences, and luminescence time decays. Moreover, the luminescence spectral feature’s dependence on the excitation energy was revealed: the UV exciton luminescence is enhanced under X-ray irradiation in all the specimens, whereas the NPs display an intriguing luminescence behavior in the VIS range where the relative intensities among the bands change according to the change in excitation energies from sub-band gap light to ionizing radiation.

In conclusion, this detailed optical analysis highlights the presence of the same five emission bands in all ZnO systems, regardless of the different synthetic provenience and size. Anyway, their relative intensities and decay times are strongly affected by the ZnO morphology and size, suggesting the possibility of varying the optical properties by choosing the dimensions, shapes and interfaces. Hence, the deep understanding of the defectiveness paves the way for the engineering of luminescent NPs with diverse optical properties, enhancing their application versatility.

Although this work focuses on ZnO systems, this approach may be potentially extended to the understanding of the defectiveness of different luminescent systems. 

## Supplementary Materials

The following are available online at https://www.mdpi.com/2079-4991/10/10/1983/s1. Details on the structural and spectroscopic characterizations of the samples are presented, together with the equations used to perform the TRPL analysis. Figure S1: XRD spectra of the nanometric and micrometric ZnO powders, Table S1: Schematic diagram of the analyzed samples.
