# Supplementary material for: Morphology Related Defectiveness in ZnO Luminescence: From Bulk to Nano-Size
Source: Nanomaterials (Basel). 2020 Oct 7;10(10):1983. doi: 10.3390/nano10101983 (PMC7601266; doi:10.3390/nano10101983)
Supplement: Supplementary file 1 [file nanomaterials-10-01983-s001.pdf]

nanomaterials

MDPI

Supporting Information for:

Morphology Related Defectiveness in ZnO Luminescence: from Bulk to Nano-Size

Roberta Crapanzano [1], Irene Villa [1*], Silvia Mostoni [2], Massimiliano D'Arienzo [2], Barbara Di Credico [2], Mauro Fasoli [1], Roberto Scotti [2], and Anna Vedda [1]

[1] Department of Materials Science, University of Milano - Bicocca, Via R. Cozzi 55, I-20125, Milano, Italy
[2] Department of Materials Science, INSTM, University of Milano - Bicocca, Via R. Cozzi 55, I-20125 Milano, Italy
* Correspondence: irene.villa@unimib.it; Tel.: +39 02 6448 5169

Samples	Size	Synthesis	Interfaces	Shape	Form and colour
**N05-HI**	5 nm	Hot Injection	Capped with stearic acid	Spherical	White powder
**N22-HI**	2 nm	Hot Injection	Capped with stearic acid	Spherical	White powder
**N05-SG-SS**	5 nm	Sol-Gel	Supported onto spherical silica NPs	Spherical	White powder
**N05-SG-SR**	5 nm	Sol-Gel	Supported onto rod-like silica NPs	Spherical	White powder
**MP**	100 nm – 2000 nm	Unknown (commercial)	Unknown (commercial)	Inhomo-geneous	White powder
**BC**	10 cm x 10 cm x 1 mm	Hydrothermal growth (commercial)	Two sides polished	Parallelepipe d	Yellow crystal

Table S1. Schematic diagram of the investigated samples.

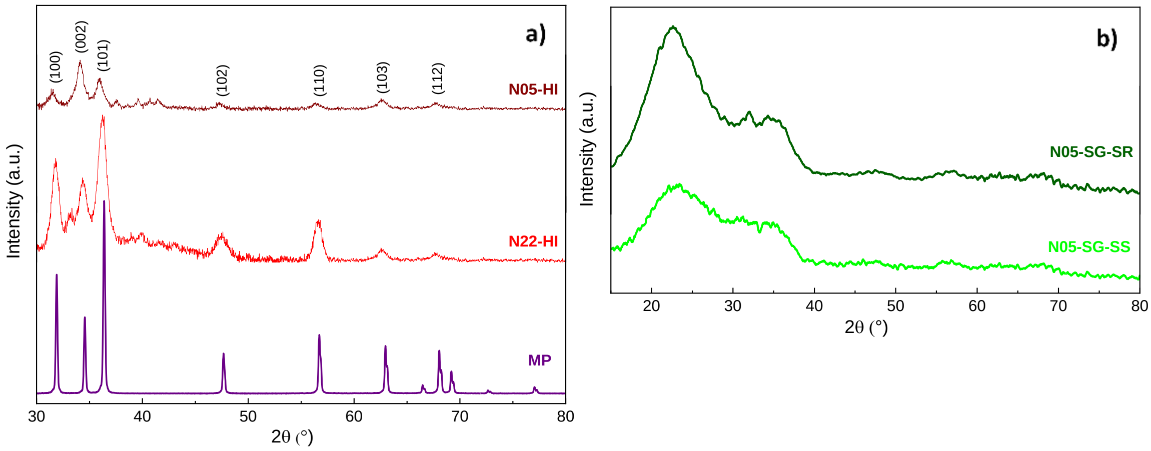

Figure S1. XRD spectra of ZnO NPs prepared by a) hot injection method (N05-HI and N22-HI, in comparison to MP) and b) supported onto SiO_2 NPs (N05-SG-SS and N05-SG-SR).

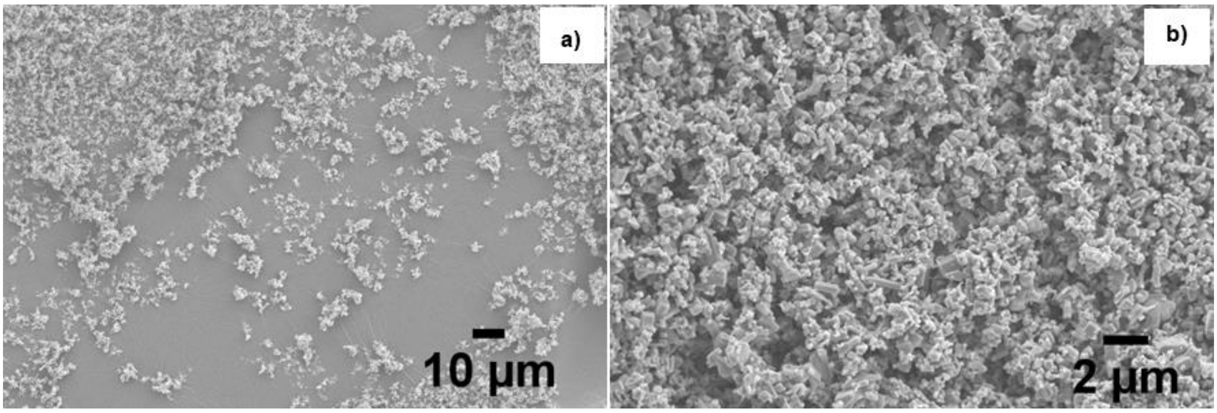

Figure S2. SEM images of MP

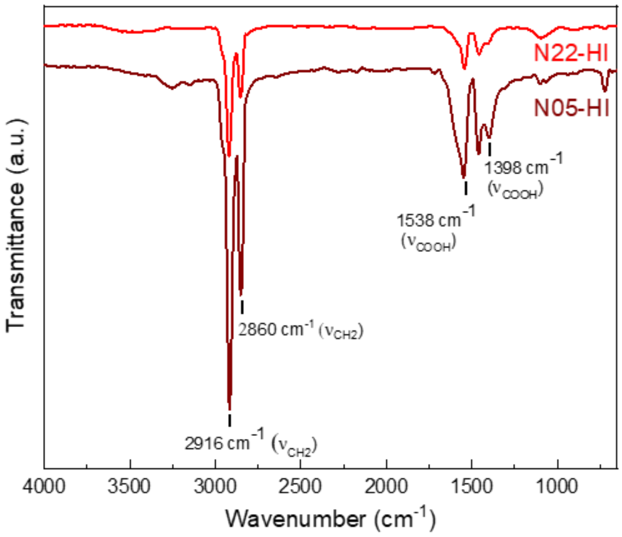

Figure S3. ATR-FTIR spectra of ZnO NPs synthesized through the hot injection method (N05-HI and N22-HI)

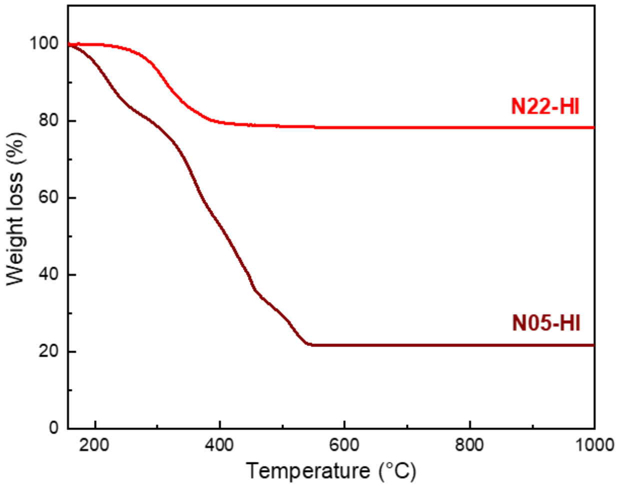

Figure S4. TGA profile of ZnO NPs prepared by hot injection. The weight loss (ΔW150-1000°C) is connected to the combustion of the surface ligands.

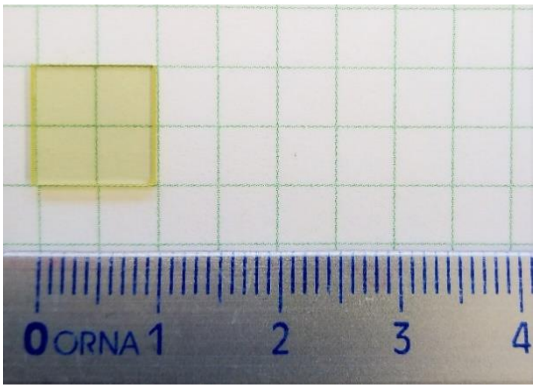

Figure S5. Photograph of commercial ZnO bulk single crystal (BC) purchased from Alineason

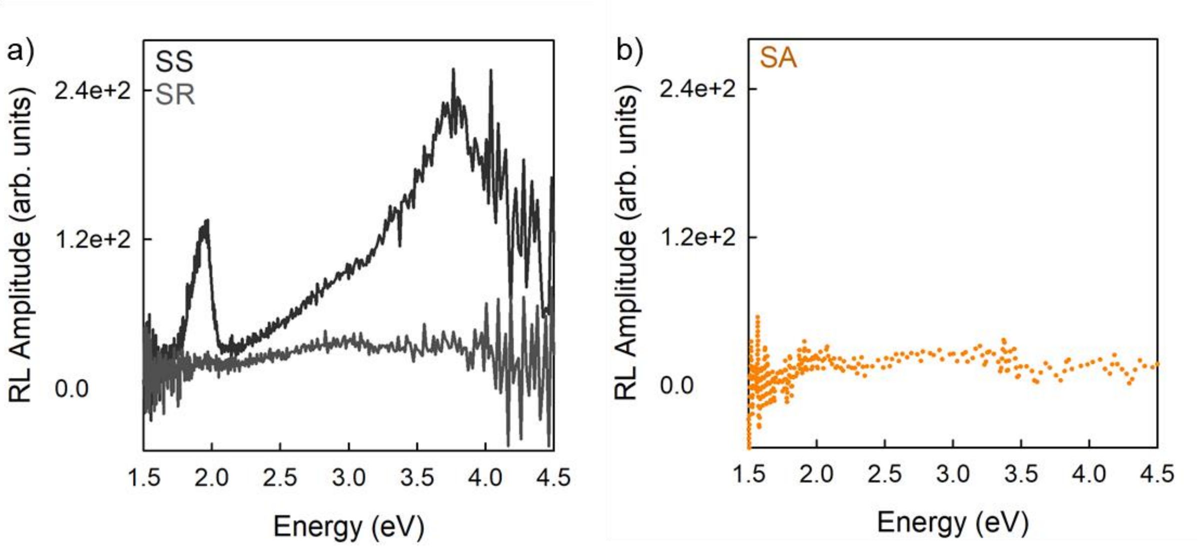

Figure S6. RL of spherical (SS) and rod-like (SR) silica NPs (a); RL of stearic acid (SA) (b)

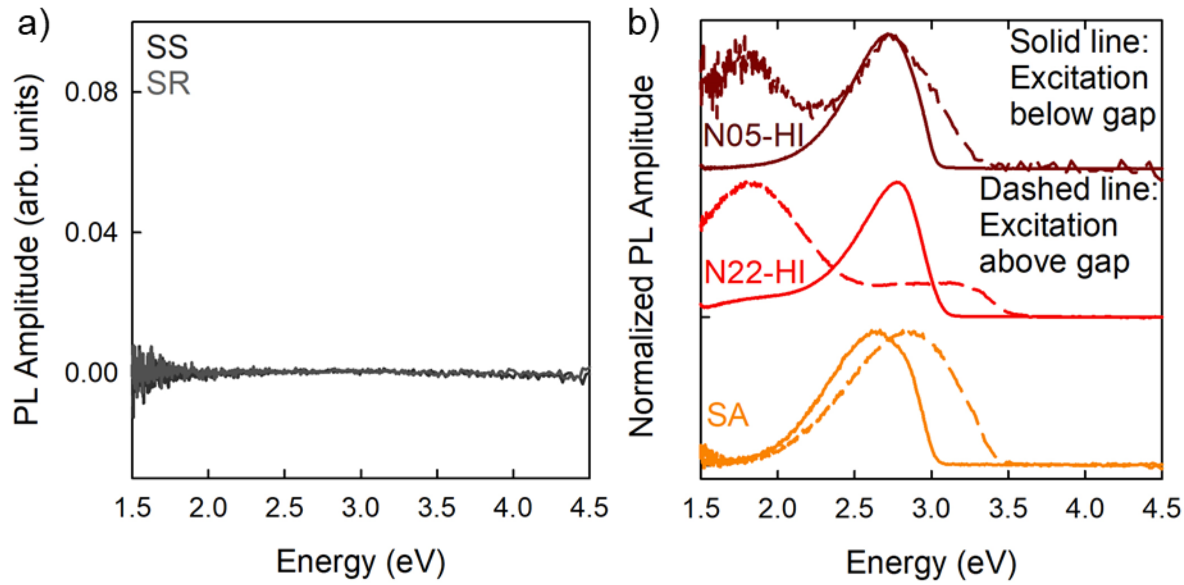

Figure S7. a) PL spectra of spherical (SS) and rod-like (SR) silica NPs measured at 3.8 eV excitation; b) PL spectra measured above (3.5-3.6 eV) and below (3.1-3.2 eV) ZnO band gap of bare ZnO NPs (N05-HI and N22-HI) and of SA.

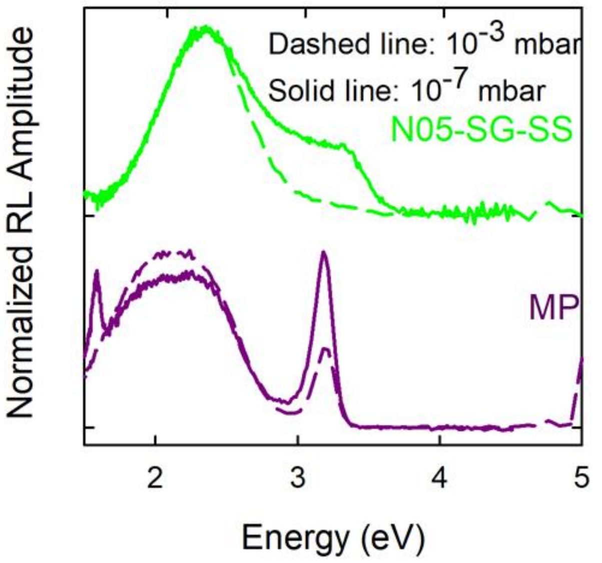

Figure S8. Normalized RL spectra of one nanometric sample (ZS) and the micrometric powder (MP) recorded at 300 K in high rotary vacuum (10^{-3} mbar, dashed line) and high vacuum (10^{-7} mbar, solid line) condition. The peak at 1.6 eV observed in the micrometric sample is caused by the diffraction second order of the exciton emission generated by the spectrograph of the detection system.

Gaussian spectral reconstruction.

RL spectra have been recorded at different temperatures from 300 K to 10 K and 10^{-7} mbar. After the collection, RL spectra have been corrected for the spectral response of the detector. All the RL spectra could be fitted in a coherent way for all the temperatures by the sum of the same Gaussian

components set reported in Table T2. To perform the fit procedure, we followed two main guidelines: 1) to use the minimum number of bands necessary to achieve a satisfactory spectrum reproduction for each sample, 2) to consider, the energy bands values reported in the literature for ZnO emissions as fit starting values, whenever possible.

The analysis of the Full Width at Half Maximum (FWHM) parameters of the Gaussian deconvolutions of RL spectra reveals that the emission bands of nanometric powders are broader than those of the micrometric sample and of the bulk one. The broadening of the bands widths in nanoscale materials can be due to the inhomogeneous structural distortions in the surroundings of optically active centers that generate a distribution of their energy level [1].

**Gaussian deconvolution parameters**		**N05-HI**	**N22-HI**	**N05-SG-SS**	**N05-SG-SR**	**MP**	**BC**
**Band A (green line)**	Energy (eV)	1.84 ± 0.02	1.73 ± 0.01	-	-	1.85 ± 0.04	1.90 ± 0.04
	FWHM (eV)	0.86 ± 0.02	0.68 ± 0.05	-	-	0.62 ± 0.03	0.56 ± 0.06
	Weight (%)	60	84	-	-	48	27
**Band B (blue line)**	Energy (eV)	-	-	2.30 ± 0.04	2.22 ± 0.01	2.33 ± 0.03	2.23 ± 0.04
	FWHM (eV)	-	-	0.78 ± 0.03	0.82 ± 0.01	0.42 ± 0.02	0.57 ± 0.06
	Weight (%)	-	-	77	92	23	45
**Band C (pink line)**	Energy (eV)	2.73 ± 0.02	2.63 ± 0.04	-	-	2.65 ± 0.06	-
	FWHM (eV)	0.63 ± 0.02	0.68 ± 0.06	-	-	0.42 ± 0.02	-
	Weight (%)	40	13	-	-	12	-
**Band D (grey line)**	Energy (eV)	-	-	2.97 ± 0.02	-	3.13 ± 0.03	-
	FWHM (eV)	-	-	0.50 ± 0.04	-	0.29 ± 0.03	-
	Weight (%)	-	-	12	-	9	-
**Band E (light blue line)**	Energy (eV)	-	3.22 ± 0.04	3.30 ± 0.04	3.23 ± 0.03	3.28 ± 0.06	3.31 ± 0.06
	FWHM (eV)	-	0.34 ± 0.02	0.36 ± 0.03	0.64 ± 0.02	0.14 ± 0.01	0.14 ± 0.02

	Weight (%)	-	3	11	8	8	28

Table S2. Fit parameters of the Gaussian deconvolution of the RL spectra of different ZnO materials. Energy and FWHM parameters of the Gaussian components have been used for all the temperatures.

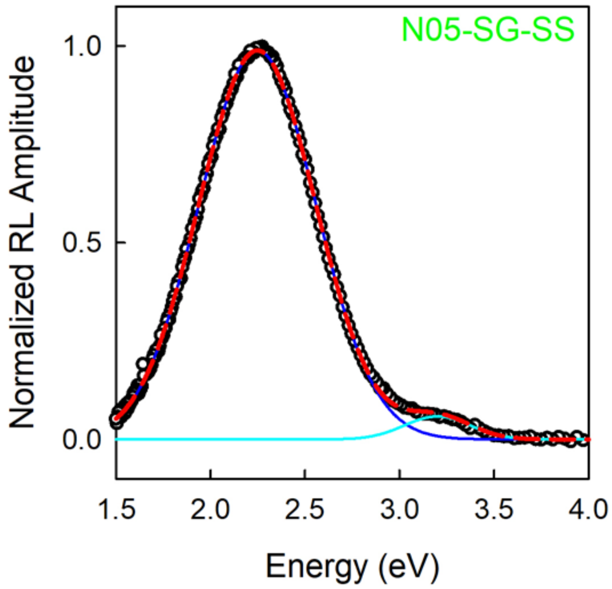

Figure S9. Deconvolution of RL emission recorded at 300 K and 10^{-3} mbar of one nanometric sample (N05-SG-SS). The fit parameters are listed in Table T1.

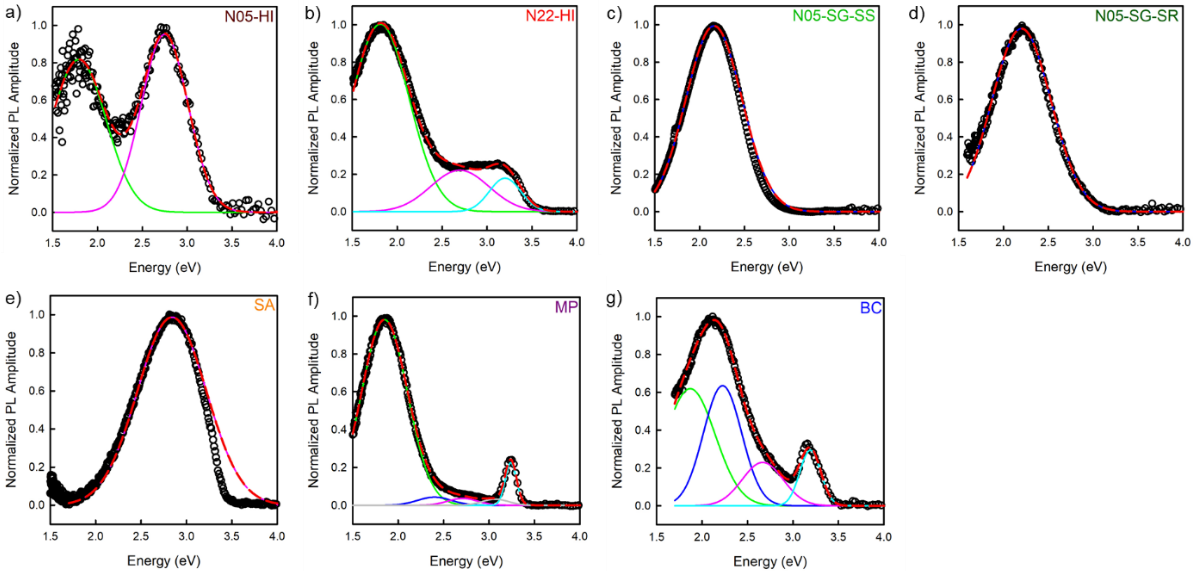

Figure S10. Gaussian deconvolution of normalized PL spectra of all ZnO samples and stearic acid (SA) recorded under CW excitation at 3.5-3.6 eV (above ZnO band gap) with the use of a long-pass filter with cutting wavelength at 370 nm (3.4 eV). Gaussian components (green, blue, pink, light blue, and grey solid lines) obtained by numerical fit are shown together with experimental curves (black

empty circle lines). The curve representing the whole numerical fit (red dashed line) is superimposed on the experimental data. The sets of all parameters of the deconvolution are listed in Tables T2 in the Supporting Information.

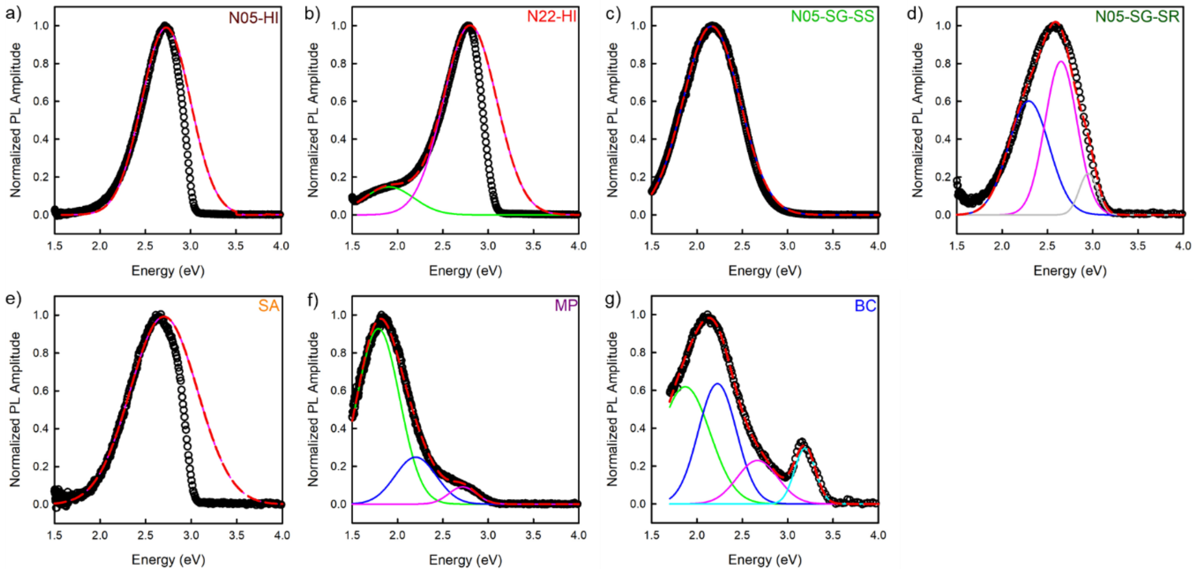

Figure S11. Gaussian deconvolution of normalized PL spectra of all ZnO samples and stearic acid (SA) recorded under CW excitation at 3.1-3.2 eV (below ZnO band gap) with the use of a long-pass filter with cutting wavelength at 418 nm (3.0 eV). Gaussian components (green, blue, pink, light blue, and grey solid lines) obtained by numerical fit are shown together with experimental curves (black empty circle lines). The curve representing the whole numerical fit (red dashed line) is superimposed on the experimental data. The sets of all parameters of the deconvolution are listed in Tables T3 in the Supporting Information.

**Gaussian deconvolution parameters**		**N05-HI**	**N22-HI**	**SA**	**N05-SG-SS**	**N05-SG-SR**	**MP**	**BC**
**Band A (green line)**	Energy (eV)	1.79 ± 0.02	1.81 ± 0.06	-	-	-	1.85 ± 0.04	1.87 ± 0.04
	FWHM (eV)	0.72 ± 0.02	0.77 ± 0.07	-	-	-	0.59 ± 0.03	0.65 ± 0.06
	Weight (%)	50	75	-	-	-	88	43
**Band B (blue line)**	Energy (eV)	-	-	-	2.16 ± 0.04	2.20 ± 0.04	2.34 ± 0.03	2.23 ± 0.04
	FWHM (eV)	-	-	-	0.74 ± 0.03	0.78	0.45 ± 0.04	0.50 ± 0.06

	Weight (%)	-	-	-	100	100	3	35
**Band C (pink line)**	Energy (eV)	2.75 ± 0.02	2.70 ± 0.07	2.84 ± 0.09	-	-	2.71 ± 0.06	2.67
	FWHM (eV)	0.62 ± 0.04	0.77 ± 0.07	0.89 ± 0.02	-	-	0.39 ± 0.02	0.53 ± 0.07
	Weight (%)	50	17	100	-	-	3	13
**Band D (grey line)**	Energy (eV)	-	-	-	-	-	3.07 ± 0.03	-
	FWHM (eV)	-	-	-	-	-	0.29 ± 0.03	-
	Weight (%)	-	-	-	-	-	2	-
**Band E (light blue line)**	Energy (eV)	-	3.20 ± 0.03	-	-	-	3.24 ± 0.05	3.19 ± 0.06
	FWHM (eV)	-	0.41 ± 0.02	-	-	-	0.12 ± 0.01	0.27 ± 0.03
	Weight (%)	-	8	-	-	-	4	9

Table S3: Fit parameters of the Gaussian deconvolution of the PL spectra of different ZnO materials and of stearic acid SA recorded exciting above ZnO band gap.

**Gaussian deconvolution parameters**		**N05-HI**	**N22-HI**	**SA**	**N05-SG-SS**	**N05-SG-SR**	**MP**	**BC**
**Band A (green line)**	Energy (eV)	-	1.90 ± 0.06	-	-	-	1.79 ± 0.04	1.85 ± 0.04
	FWHM (eV)	-	0.65 ± 0.07	-	-	-	0.55 ± 0.03	0.65 ± 0.06
	Weight (%)	-	12	-	-	-	75	39
**Band B (blue line)**	Energy (eV)	-	-	-	2.16 ± 0.04	2.29 ± 0.05	2.20 ± 0.03	2.27 ± 0.04
	FWHM (eV)	-	-	-	0.74 ± 0.03	0.53 ± 0.12	0.54 ± 0.04	0.51 ± 0.06
	Weight (%)	-	-	-	100	45	19	40

**Band C (pink line)**	Energy (eV)	2.73 ± 0.02	2.80 ± 0.07	2.70 ± 0.09	-	2.65 ± 0.05	2.72 ± 0.06	2.67
	FWHM (eV)	0.64 ± 0.04	0.69 ± 0.07	0.87 ± 0.02	-	0.42 ± 0.09	0.36 ± 0.02	0.43 ± 0.07
	Weight (%)	100	88	100	-	5	6	21
**Band D (grey line)**	Energy (eV)	-	-	-	-	2.95 ± 0.06	-	-
	FWHM (eV)	-	-	-	-	0.20 ± 0.05	-	-
	Weight (%)	-	-	-	-	47	-	-
**Band E (light blue line)**	Energy (eV)	-	-	-	-	-	-	-
	FWHM (eV)	-	-	-	-	-	-	-
	Weight (%)	-	-	-	-	-	-	-

Table S4: Fit parameters of the Gaussian deconvolution of the PL spectra of different ZnO materials and of stearic acid SA recorded exciting below ZnO band gap.

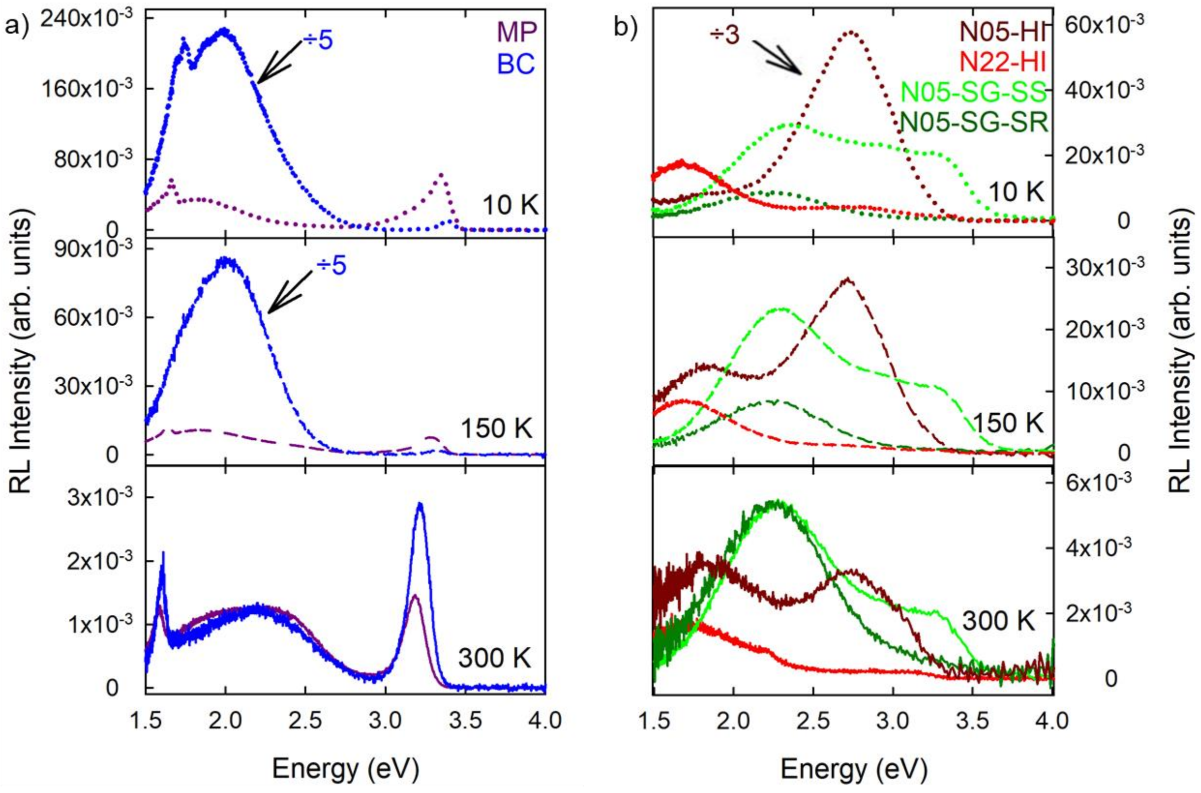

Figure S12. RL intensities recorded at 10^{-7} mbar and at 300 K (solid line), 150 K (dashed line) and 10 K (dotted line) normalized to the total area at 300 K of bulk single crystal (BC) and micrometric powder (MP) (a) and of the nanometric samples (N05-HI, N22-HI, N05-SG-SS, N05-SG-SR) (b).

Thermal activation energy.

The activation energies E_a of the thermal quenching were determined from the RL intensity dependence on the temperature (Figure 5) according to the following equation [2]:

$$I\,(T) = \frac{I_0}{1+\alpha\, e^{\frac{-E_a}{k_B T}}} \qquad \text{Equation S1}$$

where k_B is the Boltzmann's constant, α is a constant parameter, and I_0 is the luminescence intensity at 0 K. In our case, we performed the analysis on the range 100 K- 300 K and, since the intensity of the bands is approximately constant at low temperatures, for I_0 we considered the RL intensity at 50 K. In agreement with the literature [3,4] , we found a thermal activation energy of the exciton luminescence with mean value of ~40 meV for all the three systems (bulk, micrometric powder and NPs) Analyzing the defect emissions temperature dependence of the nanometric samples, we obtained activation energies of ~70 meV for band B (detected in N05-SG-SS and N05-SG-SR) and of ~40 meV for band A and of ~50 meV for band C (detected in N05-HI and N22-HI). Previous works reported similar activation energies for defect-related luminescence in ZnO nanosystems [5,6].

TRPL data analysis.

The time resolved PL spectra reported in Figure 6 show a complex behaviour. The signal decay has been reproduced with an analytically multi-exponential function:

$$I_{PL} \propto \sum_1^i A_i\, e^{-t/\tau_i} \qquad \text{Equation S2}$$

The parameters used for the fit procedure are reported below in Table 1. The weight of each component of the TRPL decay, that represents the fractional contribution, has been computed using:

$$w_i = \frac{A_i\,\tau_i}{\sum_1^i A_i\;\tau_i} \qquad \text{Equation S3}$$

where the terms $A_i\tau_i$ are proportional to the area under the decay curve for each decay time. Then, the average PL lifetime $\bar{\tau}$ has been calculated as the weighted average of the characteristic decay time for each exponential function i using:

$$\bar{\tau} = \frac{\sum_1^i w_i\,\tau_i}{\sum_1^i w_i} \qquad \text{Equation S4}$$

	**N05-HI**	**N22-HI**	**MP**	**N05-SG-SS**	**N05-SG-SR**	**N05-HI**	**N22-HI**	**SA**
	Emission energy (eV)							
	1.8 (Band A)	1.8 (Band A)	2 (Band A)	2.1 (Band B)	2.3 (Band B)	2.8 (Band C)	2.8 (Band C)	2.8 (Band C)
	Excitation energy (eV)							
	3.6	3.6	3.1	3.6	3.6	3.6	3.6	3.6
	TRPL Fit parameters							
τ_1 (ns)	1.04 ± 0.04	0.94 ± 0.04	136 ± 6	25 ± 1	10 ± 1	2.4 ± 0.1	0.85 ± 0.02	0.85 ± 0.03
$Weight_1$ (%)	45	52	4	18	33	38	87	62
τ_2 (ns)	19.2 ± 1.2	20.7 ± 2.8	934 ± 25	305 ± 6	70 ± 1	7.2 ± 0.2	4.7 ± 0.4	4.4 ± 0.2
$Weight_2$ (%)	55	48	36	21	27	62	13	38
τ_3(ns)	-	-	5102 ± 130	1282 ± 8	660 ± 4	-	-	-
$Weight_3$ (%)	-	-	60	61	40	-	-	-
y_0	-	-	0.003	-	-	-	-	-
$<\tau>$ (ns)	11 ± 1	11 ± 3	1300 ± 200	850 ± 20	286 ± 6	5.4 ± 0.3	1.5 ± 0.4	2.2 ± 0.2

Table S5. TRPL fit parameters for red (band A), green (band B) and blue (band C) emissions of the nanometric samples and the capping agent (SA) under excitation at 3.6 eV and for micrometric powder red emission (band A) under excitation at 3.1 eV.

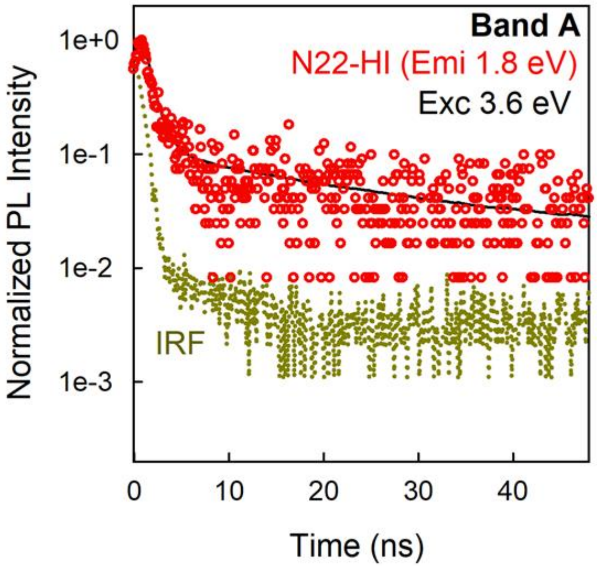

Figure S13. PL time decays of N22-HI NPs at 1.8 eV under pulsed excitation at 3.6 eV. The signal decay is fitted as a two-exponential function (black solid lines). The sets of all parameters used to model the PL decay are reported in Table 1.

Supporting Information Bibliography:

1. Solé, J.G.; Bausá, L.E.; Jaque, D. *An Introduction to the Optical Spectroscopy of Inorganic Solids*; John Wiley & Sons, Ltd: Chichester, UK, 2005; ISBN 9780470016046.

2. Williams, F.E.; Eyring, H. The mechanism of the luminescence of solids. *J. Chem. Phys.* **1947**, *15*, 289–304, doi:10.1063/1.1746499.

3. Kumar, N.; Kaur, R.; Mehra, R.M. Photoluminescence studies in sol-gel derived Zno films. *J. Lumin.* **2007**, *126*, 784–788, doi:10.1016/j.jlumin.2006.11.012.

4. Fonoberov, V.A.; Alim, K.A.; Balandin, A.A.; Xiu, F.; Liu, J. Photoluminescence investigation of the carrier recombination processes in ZnO quantum dots and nanocrystals. *Phys. Rev. B - Condens. Matter Mater. Phys.* **2006**, *73*, 1–9, doi:10.1103/PhysRevB.73.165317.

5. Chen, H.I.; Hsiao, J.J.; Fang, C.H.; Jiang, J.A.; Wang, J.C.; Wu, Y.F.; Nee, T.E. Investigation of the thermal photocarrier interaction–recombination in ZnO nanostructures fabricated by the hydrothermal method. *J. Lumin.* **2017**, *190*, 136–140, doi:10.1016/j.jlumin.2017.05.020.

6. El Filali, B.; Torchynska, T. V.; Polupan, G.; Shcherbyna, L. Radiative defects, emission and structure of ZnO nanocrystals obtained by electrochemical method. *Mater. Res. Bull.* **2017**, *85*, 161–167, doi:10.1016/j.materresbull.2016.09.016.
